# Supplementary material for: Experiences of violence and mental health outcomes among Asian American transgender adults in the United States
Source: PLoS One. 2021 Mar 4;16(3):e0247812. doi: 10.1371/journal.pone.0247812 (PMC7932064; doi:10.1371/journal.pone.0247812)
Supplement: Attachment — Submitted filename: PONE-D-20-21733.docx [file pone.0247812.s001.docx]

PLOS One

Article ID: **PONE-D-20-21733**

Title: Experiences of violence and mental health outcomes among Asian American

transgender adults in the United States

Comments to authors

The manuscript, “Experiences of violence and mental health outcomes among Asian American transgender adults in the United States,” describes a study examining experiences of Asian American transgender adults in terms of violence and mental health using the 2015 US Transgender Survey. Key findings included high rates of minority stress (discrimination and abuse) as well as negative mental health outcomes, such as suicide ideation and distress. Authors highlight the need for reducing exposure to negative outcomes and greater use of trauma informed care approaches in healthcare settings. Several strengths of this study include novel topic and potential for future research. Despite several strengths, there are a number of research and conceptual issues that if addressed, would significantly improve the manuscript. These are explained below in no particular order.

1. It would be beneficial to readers to learn more about potential research on mental health of Asian American transgender and additional gender minority populations. For instance, several articles should be considered:

Ching TH, Lee SY, Chen J, So RP, Williams MT. A model of intersectional stress and trauma in Asian American sexual and gender minorities. Psychology of violence. 2018 Nov;8(6):657.

Operario D, Nemoto T. Sexual risk behavior and substance use among a sample of Asian Pacific Islander transgendered women. AIDS Education & Prevention. 2005 Oct 1;17(5):430-43.

Snow A, Cerel J, Loeffler DN, Flaherty C. Barriers to mental health care for transgender and gender-nonconforming adults: a systematic literature review. Health & social work. 2019 Aug 2;44(3):149-55.

Howard SD, Lee KL, Nathan AG, Wenger HC, Chin MH, Cook SC. Healthcare experiences of transgender people of color. Journal of general internal medicine. 2019 Oct 1;34(10):2068-74.

2. Methods: Consider providing Cronbach alpha for the Kessler-6 scale for study sample.
3. Table 2 needs a more descriptive/detailed title.
4. It appears that chi-square tests were run to test potential associations. If this represents potential prevalence by factors that are weighted, authors might consider putting the 95% Confidence Intervals
5. For logistic regression, authors may want to present findings in terms of prevalence ratios.
6. Did authors consider testing any interactions with demographics (age, education level, employment, poverty) as it appears these could represent intersectionality. Also may want to consider differences by gender identity (e.g., non-binary vs. gender male/female identifying groups).
7. It appears that additional limitations of the 2015 USTS survey were not mentioned. Authors should consider additional concerns in terms of nonprobability sample. See the following for additional limitations:

Henderson, E.R., Blosnich, J.R., Herman, J.L. and Meyer, I.H., 2019. Considerations on sampling in transgender health disparities research. LGBT health, 6(6), pp.267-270.

8. The discussion needs further development, such as other studies in cisgender sexual minorities and cisgender Asian Americans with regards to experiencing abuse and risk of suicide/mental health distress.
9. Discussion should touch how healthcare needs and tailoring of trauma-informed care that considers intersectionality, such as identifying as both Asian American and a gender minority.
10. It might be helpful to readers to further tease of unique cultural concerns for Asian American gender minorities as well as details on how research can be more inclusive of these communities. It might be helpful to expand on any findings in terms of care and needed research on immigration/acculturation and gender minorities.
