# Supplementary material for: Experiences of violence and mental health outcomes among Asian American transgender adults in the United States
Source: PLoS One. 2021 Mar 4;16(3):e0247812. doi: 10.1371/journal.pone.0247812 (PMC7932064; doi:10.1371/journal.pone.0247812)
Supplement: Attachment — Submitted filename: Response to Reviewers-12-6-2020.docx [file pone.0247812.s002.docx]

Response to reviews:

Thank you for the comprehensive feedback that allows our manuscript to be improved. We have addressed each of the comments below, added content in the manuscript/tables, and have marked them as track changes as well. We have bolded the responses below for ease of finding.

**Review 1**

1. It would be beneficial to readers to learn more about potential research on mental health of Asian American transgender and additional gender minority populations. For instance, several articles should be considered:

Ching TH, Lee SY, Chen J, So RP, Williams MT. A model of intersectional stress and trauma in Asian American sexual and gender minorities. Psychology of violence. 2018 Nov;8(6):657.

Operario D, Nemoto T. Sexual risk behavior and substance use among a sample of Asian Pacific Islander transgendered women. AIDS Education & Prevention. 2005 Oct 1;17(5):430-43.

Snow A, Cerel J, Loeffler DN, Flaherty C. Barriers to mental health care for transgender and gender-nonconforming adults: a systematic literature review. Health & social work. 2019 Aug 2;44(3):149-55.

Howard SD, Lee KL, Nathan AG, Wenger HC, Chin MH, Cook SC. Healthcare experiences of transgender people of color. Journal of general internal medicine. 2019 Oct 1;34(10):2068-74.

**Response: Our second paragraph addressed the current literature (peer-reviewed and others) on mental health burden Asian American sexual and gender minorities. We have further expanded it by including additional references from those noted above. They have been included in the introduction section (Ching et al., Operaio and Nemoto, Snow et al.) as well as in the discussion section (Howard et al.), in addition to a few others.**

2. Methods: Consider providing Cronbach alpha for the Kessler-6 scale for study sample.

**Response: The Cronbach alpha of .89 has been added in the methods section.**

3. Table 2 needs a more descriptive/detailed title.

**Response: We have added a more descriptive title that provides details on the context (including analysis) of the table results.**

4. It appears that chi-square tests were run to test potential associations. If this represents potential prevalence by factors that are weighted, authors might consider putting the 95% Confidence Intervals

**Response: The weights applied in this study were used to reduce bias for demographic representation. Since this was a non-probability sample, confidence intervals may not be an appropriate measure of reliability since their estimates are inherently biased from non-random sampling. We have stated the limitations of a non-probability sample in our discussion.**

5. For logistic regression, authors may want to present findings in terms of prevalence ratios.

**Response: We did consider reporting prevalence ratio, but during the literature review, we noted that majority of the studies used odds ratio. In order to be utilized for comparison purposes, as well as putative meta analysis by other researchers, we wanted to remain consistent. The interpretation, however, does provide better context on the data.**

6. Did authors consider testing any interactions with demographics (age, education level, employment, poverty) as it appears these could represent intersectionality. Also may want to consider differences by gender identity (e.g., non-binary vs. gender male/female identifying groups).

**Response:** **We checked interactions for based on relevant literature and it is noted in the methods. Survey participants were informed that “trans” or “transgender” is defined as all trans and non-binary identities for the purposes of the survey. As such, the data is inclusive of the different identities. However, per the recommendation, we have added a sub-analysis in our study as part of Tables 4 and 5.**

7. It appears that additional limitations of the 2015 USTS survey were not mentioned. Authors should consider additional concerns in terms of nonprobability sample. See the following for additional limitations:

Henderson, E.R., Blosnich, J.R., Herman, J.L. and Meyer, I.H., 2019. Considerations on sampling in transgender health disparities research. LGBT health, 6(6), pp.267-270.

**Response: We have included content from this study as well as additional to address the limitations.**

8. The discussion needs further development, such as other studies in cisgender sexual minorities and cisgender Asian Americans with regards to experiencing abuse and risk of suicide/mental health distress.

**Response: We included cisgender focused studies in introduction and discussion, but limited to ensure the focus remains on the need for such among the SGM population, especially Asian Americans transgender populations.**

9. Discussion should touch how healthcare needs and tailoring of trauma-informed care that considers intersectionality, such as identifying as both Asian American and a gender minority.

**Response: We have added context related to such analysis in the discussion along with the following comment’s content as well.**

10. It might be helpful to readers to further tease of unique cultural concerns for Asian American gender minorities as well as details on how research can be more inclusive of these communities. It might be helpful to expand on any findings in terms of care and needed research on immigration/acculturation and gender minorities.

**Response: We have added context related to addressing the unique cultural factors in the discussion.**

**Review 2**

1. Please specify the number of participants included in your sample for analysis - this currently only to be provided as 7.89%

**Response: We have added the sample in the results section.**

2. Please add more information on how the survey was delivered (online? face to face?), how were participants recruited? What was the eligibility criteria (who was targeted)?

**Response: This has been added in the data source section of the methods.**

3. I have some issues with how the outcomes were defined; the  authors state that the outcome variables include dichotomized versions of suicidal ideation, suicidal thoughts, and serious psychological distress - however, only suicide thoughts are reported on (not ideation) - how do thoughts and ideation differ in this study (they usually mean the same thing)? This requires further clarification.

Moreover, what instruments were used to ask participants about suicidal ideation and attempts? Were they scales, were they validated? If they were scales, I would query why the authors recoded them into dichotomous variables when a continuous score would have been clinically more significant. If authors did recode scales into dichotomous variables, how was the decision made as to what constitutes a 'yes' answer and what is a 'no' answer? Much more detail is needed.

**Response: We agree that more detailed content would be valuable. However, the USTS data provides the dichotomized variables as utilized in this study and others. However, the information on instruments for suicidal thoughts, suicidal attempts, and SPD, are added in the measures section.**

4. I similarly question the authors decision to create over simplistic 'yes/no' variables to assess experience of violence, when there appears to be rich data on different types of violence; there is literature to support that different types of violence have different impacts on mental health, and it would be a genuine value add of this study to examine the experience of different types of violence on suicide outcomes in this transgender population. I would strongly suggest some re analysis to provide a more nuanced understanding of the effects of types of violence on suicidal thoughts and attempts. 

**Response: USTS has four types of abuses included: past year verbal harassment, past year physical attack, past year unwanted sexual contact, and ever experience of unwanted sexual contact. While we acknowledge that verbal versus physical versus sexual can have differing outcomes, our goal was to explore any experiences of violence and thus we categorized them as any abuse. With a larger sample size, further disaggregation may be of value. However, we did add additional types of abuse, including partner and harassment during bathroom use.**
