# Supplementary material for: Experiences of violence and mental health outcomes among Asian American transgender adults in the United States
Source: PLoS One. 2021 Mar 4;16(3):e0247812. doi: 10.1371/journal.pone.0247812 (PMC7932064; doi:10.1371/journal.pone.0247812)
Supplement: Attachment — Submitted filename: Response to Reviewers-2-8-2021.docx [file pone.0247812.s003.docx]

Response to reviews:

**Thank you for the reviews of our manuscript. We have addressed all the reviews and responses are noted below in bold, for ease of finding.**

Abstract
• Methods: Can you identify how you subsetted to Asian Americans? How was this assessed in the 2015 USTS? Who is included in this ethnic group?
**Response: We included this in the methods section of the manuscript, instead of abstract, to ensure we remain within the word limit. However, we added a statement to clarify it was census defined as well.**

• Methods/Results: Improve notation for aOR and 95% CI for consistency. For example, define the format first/once in the methods then use it consistently in the results. For example:
o Methods: Adjusted odds ratio and 95% confidence interval (aOR; 95% CI) for each outcome will be adjusted for age, marital status, citizenship status, education level, employment status, and poverty level.

o Results: Experience of harassment/abuse during bathroom use had increased odds of suicidal attempts (aOR: 1.81; 95% CI: 1.41, 2.31).

**Response: We have updated the methods section in the abstract accordingly.**
**We have updated the results section as well to be consistent and note aOR in each case.**

Manuscript
• Line 59-60: Does the literature refer to diagnosed cases or self-report?
• Can you speak to stigma for seeking mental health services and care?
• Line 70-72: same question as above. Does the literature refer to diagnosed cases or self-report?
**Response: This has been updated in the instruction section. We also added the importance of addressing stigma in the conclusion.**

• Line 155 and 157: Why is “OR” in caps? Perhaps consider listing the measures without stating the entire question asked, separated with semicolons. For example, instead of “In the past year, did anyone verbally harass you for any reason?”, you can indicate “verbal harassment for any reason in past year”.

**Response: In the previous revision cycle, we were asked to list the full question. However, we have updated the structure to read with more ease and not capitalized the OR.**

• Line 211: Improve the statement for alpha or significance level. It is not clear what you are denoting. Perhaps you can say that “The significance level of .05 was used” or “A p-value less than 0.05 determined a statistically significant difference”. You use other significance levels in the tables. You should mention these here as well.

**Response: We’ve updated this section to read more clearly as well as add the .01 and .001 mentioned in the tables.**

• Methods/Results: as indicated for the abstract, improve notation for aOR and 95% CI for consistency. For example, define the format first/once in the methods then use it consistently in the results.

**Response: We have updated to be consistent and note aOR in each case.**

• Results: you are referring to “significantly higher “rates. Did you conduct one-tail or two tailed statistical tests? Your method section does not indicate the bivariate tests used (Line 200).

**Response: We added a statement on using two-tailed in the data analysis section of the methods. We also added survey-weighted chi-square for bivariate analysis in the methods section.**

• Results: Be consistent with rounding for percentages reported though this section including tables.

**Response: We have updated to remain consistent.**

• Results: In tables, improve title and include sample sizes in the title or somewhere in the table. Distinguish headers from variables. Perhaps consider moving the asterisks denoting significant findings.

**Response: We have updated the tables though moving the asterisks may not reflect the significance of the cell. However, we have tabbed the variables to make it more clear.**

• Tables: Consider justifying to the right for the numeric columns.

**Response: They have been updated to be justified to the right.**

• Table 2: You have a symbol in front of “~U.S. Citizen” that you did not define at the footer of the table.

**Response: This was a typo that has been now deleted.**

• Conclusion: this section lacks detail particularly to what the results indicate. What type of clinicians are your referring to? What type of assessments are your referring to?

**Response: We have updated the conclusion to clarify these items.**
